# Bridging perfectionism and innovation—a moderated-mediation model based on achievement goal theory

**DOI:** 10.3389/fpsyg.2025.1468489

**Published:** 2025-03-26

**Authors:** Yingya Jia, Shuping Yue, Manci Fang, Xiaoyu Wang

**Affiliations:** ^1^School of Management, Shanghai University, Shanghai, China; ^2^School of Management, Innovation and Entrepreneurship Research Center, Shanghai University, Shanghai, China; ^3^Harrow International School Hong Kong, Tuen Mun, Hong Kong SAR, China; ^4^School of Public Management, East China Normal University, Shanghai, China

**Keywords:** employee perfectionism, innovation performance, learning goal orientation, perceived failure analysis, achievement goal theory

## Abstract

**Introduction:**

There exists a significant gap in the existing literature regarding how and when employee perfectionism impacts innovation performance. This study aims to fill this gap by exploring the relationship between employee perfectionism and innovation performance.

**Methods:**

Grounded in achievement goal theory, a moderated-mediation model is proposed. In this model, learning goal orientation acts as a mediator for the distinct influences of negative and positive perfectionism on innovation performance, and perceived failure analysis serves as an organizational culture to moderate this mediation. Survey data from 378 core R&D employees in nine high-tech manufacturing firms in China were collected for analysis.

**Results:**

The findings reveal that negative perfectionism hinders innovation performance as it fosters avoidance behaviors and risk aversion. On the contrary, positive perfectionism enhances innovation performance by promoting mastery-oriented learning and experimentation. Moreover, perceived failure analysis strengthens the link between positive perfectionism and innovation performance by validating mastery goals and encouraging learning from failures. However, it has no significant effect on the negative perfectionism pathway, since employees with an avoidance orientation perceive failure analysis as a threat rather than a source of useful information.

**Discussion:**

These results contribute to the literature in three aspects. Firstly, they highlight the dual nature of perfectionism in innovation settings. Secondly, they emphasize the mediating role of learning goal orientation in converting perfectionistic tendencies into innovation outcomes. Thirdly, they identify perceived failure analysis as a cultural factor that amplifies the benefits of adaptive perfectionism but fails to mitigate the risks of maladaptive perfectionism. The study also offers practical insights for organizations to utilize the adaptive potential of perfectionism while minimizing its negative impacts.

## 1 Introduction

Perfectionists are often described as individuals who strive for excellence in every aspect of their lives (Stoeber and Stoeber, 2009). Employees exhibiting high levels of perfectionism often establish exceptionally elevated standards, pursue flawlessness, and critically evaluate their own performance, which may lead them to experience an increased occurrence of negative emotions (Flett and Hewitt, 2002). Previous studies have shown that perfectionistic employees are overly self-critical, a trait that is positively associated with stress and burnout (Stoeber and Childs, 2010); this self-criticism can negatively impact performance through emotional exhaustion (Pindek, 2020). Conversely, some researchers have examined perfectionism from a positive perspective. For example, Adler proposed that striving for perfection serves as an intrinsic motivator for self-development, encouraging individuals to adapt to their environment and pursue improvement. Building on this perspective, studies indicate that highly perfectionistic individuals actively pursue change when generating work-related ideas. On days when their perfectionistic efforts are activated, employees focus on achieving their high-performance standards and intentionally work toward their goals, which is expected to facilitate to goal progress (Mohr et al., 2022). This seemingly contradictory evidence may stem from treating perfectionism as a single construct without distinguishing its dimensions. Therefore, the following questions remain for us to answer: First, how do different dimensions of employee perfectionism influence innovation performance? Second, how do organizational factors influence the process aforementioned?

A few studies have investigated the dual dimensions of perfectionism (Hill et al., 2004; Shafran and Mansell, 2001). For instance, Harari et al. (2018) reviewed research demonstrating that employee perfectionism influences innovation performance in two distinct ways: adaptive perfectionism, which is associated with excellence-seeking behaviors, and maladaptive perfectionism, which is linked to failure-avoidance behaviors. Similarly, Haase and Prapavessis (2004) categorize perfectionism into positive and negative forms. On the one hand, positive perfectionism is characterized by individuals’ tendency to set and pursue exceptionally high standards, demonstrating a commitment to thoroughness and precision in task completion (Flett and Hewitt, 2002). This form of perfectionism can enhance innovation performance by motivating individuals to strive for excellence in their work. On the other hand, negative perfectionism often leads to increased demands and stress (Flaxman et al., 2012), as individuals focus on avoiding failure, which can result in negative outcomes such as performance anxiety and depression (Haase and Prapavessis, 2004). These adverse effects may, in turn, impede innovation performance. Therefore, perfectionism has generally been conceptualized as a motivational trait in previous research (Harari et al., 2018; Hewitt et al., 2017), which relates to employees’ innovation performance. While previous studies have examined the relationship between perfectionism and innovation (Harari et al., 2018; Hewitt and Flett, 1991), they often conflate adaptive and maladaptive dimensions or fail to consider the mediating role of motivational processes.

In this study, we introduce a distinctive integration of achievement goal theory with a multidimensional perspective on perfectionism (Hill et al., 2004). Our central proposition is that learning goal orientation serves as a key mediator, clarifying how the dual dimensions of perfectionism exert different impacts on innovation. Moreover, we introduce perceived failure analysis as a cultural moderator, exploring the conditions in which these effects are either intensified or diminished.

Rooted in achievement goal theory (Urdan and Kaplan, 2020), we assert that motivational factors play a crucial role in explaining individual differences in innovation performance (Hirst et al., 2009; Pintrich, 2000a; van Yperen and Janssen, 2002). Achievement goal theory suggests that individuals adopt distinct goal orientations, which shape their motivation and behavior in achievement-related settings. Specifically, it differentiates between mastery (learning) goals, which emphasize self-improvement and skill acquisition, and performance goals, which focus on demonstrating competence in relation to others (Pintrich, 2000a). Notably, these goal orientations are not merely simple behavioral objectives but rather represent complex cognitive frameworks that incorporate beliefs about competence, effort, and tolerance for errors (Pintrich, 2000a; Urdan and Kaplan, 2020). Building on the distinction between mastery and performance goals, our study associates positive perfectionism with a mastery-oriented cognitive schema, defined by a strong drive for self-improvement. In contrast, negative perfectionism is linked to a performance-avoidance schema, primarily focused on failure avoidance (Elliot and McGregor, 2001; Pintrich, 2000a). This theoretical connection explains how positive perfectionism can enhance learning goal orientation—a process driven by mastery motivation—while negative perfectionism inhibits it. Furthermore, we explore the concept of perceived failure analysis, an aspect of organizational culture that assesses how actively and intentionally employees in a firm analyze the mechanisms and causes of past failures (Danneels and Vestal, 2020; Denrell, 2003; Edmondson, 2004; Tjosvold et al., 2004). We also examine its moderating effects on the initial-stage individual motivational processes.

This study presents a novel and clear theoretical framework regarding the relationship between employee perfectionism and innovation performance. First, we explore the multidimensional nature of perfectionism and its dual effects on innovation, highlighting the “double-edged sword” of perfectionism. More importantly, we provide a clearer mechanism for understanding how employee perfectionism influences innovation performance by introducing one of the motivational factors as the mediator in our model, that is, learning goal orientation (Harari et al., 2018; Hrabluik et al., 2012; Locander et al., 2015). Second, we discover that perceived failure analysis as an organizational culture would have different influences on the relationship between employee perfectionism and innovation performance through different mechanisms. This insight can enhance our understanding of how organizational factors influence the innovation process of employees (Danneels, 2008; Danneels and Vestal, 2020; McGrath, 1999; Shepherd et al., 2011). Third, according to achievement goal theory, we introduce a personality relevant to innovation (i.e., perfectionism) and discuss its role in an individual motivational process (Lee et al., 2003; Sorić et al., 2017; Steinmayr and Spinath, 2008), which can provide further insights into the differences in employees’ innovation performance.

## 2 Theory and hypotheses

### 2.1 The concept of perfectionism

In this study, we adopt a two-factor structure of perfectionism, that is, negative perfectionism and positive perfectionism (Haase and Prapavessis, 2004). Based on Haase and Prapavessis (2004), we define negative perfectionism as the tendency to have strong motivation to achieve a specific goal while emphasizing the avoidance of adverse outcomes and positive perfectionism as the tendency to have strong motivation to achieve a specific goal while focusing on the achievement of favorable outcomes.

Although both of these concepts capture the tendency of individuals to achieve specific goals or outcomes, their focus on the outcomes is different. People high on negative perfectionism tend to have more pessimistic attitudes. They would attempt to avoid the failure to achieve specific goals and the adverse outcomes brought by that failure. In contrast, people high on positive perfectionism tend to have more optimistic attitudes and focus more on the achievement obtained and the favorable outcomes when realizing specific goals. The distinction between negative perfectionism and positive perfectionism is grounded in behavioral theory (Skinner, 1968), under which theoretical framework a similar behavior is likely to elicit different emotional responses, depending on whether it is a function of negative or positive reinforcement. Thus, we argue that, in the organizational innovation process, where failures occur frequently, the distinctions between the two dimensions of perfectionism would be salient (Harari et al., 2018), which can significantly impact innovation performance.

Few studies have categorized perfectionism. For example, Hill et al. (2004) created the Perfectionism Inventory with eight scales, covering self-evaluation, relationships, and thought processes. Shafran and Mansell (2001) reviewed perfectionism, breaking it into dimensions such as self-, other- and socially- prescribed perfectionism or those related to mistakes, standards, and parental influence. Their article highlighted the construct’s complexity and the need for better assessment. However, a majority of research have discussed a lot about the relationship between perfectionism and other work-related constructs, for instance, engagement, workaholism, burnout, stress, anxiety (Childs and Stoeber, 2012; Cole et al., 2012; Harari et al., 2018; Hill and Curran, 2016), while these literatures have paid limited attention to the full effect of perfectionism (Ozbilir et al., 2015), especially to the double-edged sword effect of employee perfectionism on innovation performance, which can be explained by the multifaced characteristics of perfectionism. We focus on the double-edged sword effect of employee perfectionism and develop a model to discuss its relationship with innovation performance through the individual motivational processes while also considering organizational factors.

### 2.2 Employee perfectionism and innovation performance

The innovation process involves developing and selecting ideas for innovation and then transforming these ideas into innovation. This process is characterized as risky and uncertain (Edmondson and Nembhard, 2009), because novelty is central to innovation, which also implies high ambiguity and failure rate (Bowers and Khorakian, 2014; Danneels and Vestal, 2020). Therefore, the attitudes that employees have toward the achievement of specific goals or outcomes are closely related to how employees react toward the activities during the innovation process, thus influencing their innovation performance. According to achievement goal theory, mastery-oriented individuals prioritize skill development and view challenges as opportunities for growth (Dweck, 1999). Positive perfectionism, with its emphasis on attaining high standards through effort, aligns with this mastery orientation, thereby fostering innovation. Conversely, performance-avoidance goals (associated with negative perfectionism) trigger anxiety about failure, leading to risk aversion and reduced exploratory behaviors critical for innovation (Pintrich, 2000b). Next, we will discuss in detail why these two dimensions of perfectionism affect innovation performance differently.

As grounded in achievement goal theory, negative perfectionism is negatively correlated with innovation performance, primarily for the following three reasons. According to this theory, individuals with performance-avoidance goals—those focused on avoiding failure rather than achieving success—tend to exhibit behaviors and mindsets that can hinder innovative outcomes. First, employees with negative perfectionism have a strong inclination to avoid failure and its potential negative consequences (Haase and Prapavessis, 2004). This failure-avoidance orientation makes them less inclined to take risks due to fear of organizational punishment (Danneels, 2008; Danneels and Vestal, 2020; Delbecq and Mills, 1985; Maidique and Hayes, 1984; McGrath, 1999), which consequently limits their engagement in innovative activities (García-Granero et al., 2015). Second, setting excessively high standards can increase their exposure to negative emotions, such as shame, sadness, and stress, especially if they do not meet performance expectations or encounter setbacks in the innovation process (Dunkley et al., 2003; Stoeber and Otto, 2006; Eggers, 2012; Khanna et al., 2016). To resume innovative efforts, these individuals must first recover from negative emotions that disrupt their focus and engagement (Shepherd et al., 2011). Third, the high goals associated with negative perfectionism are inherently challenging, demanding more effort and yielding lower chances of success compared to more attainable goals (Erez and Zidon, 1984). This decreased likelihood of achievement can diminish employees’ commitment to innovation goals, ultimately impacting their innovation performance (Locke and Latham, 2002).

Positive perfectionism correlates positively with innovation performance, primarily for the following two reasons. According to achievement goal theory, individuals with a mastery-oriented goal focus on self-improvement, skill development, and achieving high standards, which can drive behaviors conducive to innovation. First, employees who are highly positive in perfectionism are typically strongly motivated to achieve specific goals and place a high value on attaining favorable outcomes (Haase and Prapavessis, 2004). This goal-focused orientation fosters a willingness to take calculated risks, driven by a desire to experience the fulfillment that comes from achieving challenging goals (Spector, 1956). Such risk-taking behavior is associated with enhanced innovation performance, encouraging exploration and experimentation (García-Granero et al., 2015). Second, setting high goals tends to increase employees’ engagement with their work, motivating them to strive toward these ambitious targets (Green et al., 2017; Locke and Latham, 2002). This heightened engagement and commitment to goal attainment can enhance their focus and perseverance, factors that are crucial for innovation and continuous improvement. Thus, we propose:

*Hypothesis 1a: Employees’ negative perfectionism is negatively related to their innovation performance.*

*Hypothesis 1b: Employees’ positive perfectionism is positively related to their innovation performance.*

### 2.3 Employee perfectionism and learning goal orientation

According to achievement goal theory, an individual’s goal orientation forms a framework for how he/she approaches, experiences, and responds to different achievement situations (Barron and Harackiewicz, 2000; Dweck, 1999; Nicholls, 1984; Pintrich, 2000b; van Yperen and Janssen, 2002). The innovation process has significant characteristics of risks and uncertainty (Edmondson and Nembhard, 2009), where the intrinsic motivation to achieve the goals orienting the innovative activities is important (Hirst et al., 2009). In this study, we focus on one of the goal orientations, that is, learning goal orientation, which refers to the individual’s tendency to develop competence through seeking challenges, acquiring new skills, mastering new situations, and learning from experience (Dweck, 1986; Dweck and Leggett, 1988; Vande Walle, 1997). Employees exhibiting various tendencies across different aspects of perfectionism are likely to hold differing attitudes toward developing competence during the innovation process, which indicates that employee perfectionism influences their learning goal orientation.

Negative perfectionism is negatively correlated to learning goal orientation in three ways. First, innovation is a recurring process of experimentation and learning (Thomke, 2003), where employees are more likely to be exposed to failure (Danneels and Vestal, 2020). Owing to the fear of failing to achieve their goals and adverse outcomes brought by failure (Haase and Prapavessis, 2004), negative perfectionistic employees would have lower willingness to implement experimentation during the innovation process and learn from failure, primarily to avoid experiencing feelings of shame, sadness, stress and self-worthlessness (Dunkley et al., 2003; Eggers, 2012; Harari et al., 2018; Khanna et al., 2016; Shepherd et al., 2011; Stoeber and Otto, 2006). Second, the setting of higher goals usually means that they are more challenging to achieve, require higher effort, and are associated with lower chances of success than lower goals (Erez and Zidon, 1984), which can bring about strong feelings of frustration and decrease their commitment to the attainment of innovation goals (Locke and Latham, 2002), which would lower the employees’ tendencies of learning from failure, owing to the exhaustion of motivation to achieve their goals (Rubino et al., 2009; Skaalvik and Skaalvik, 2016). Third, employees with negative perfectionism are more frequently exposed to negative emotions such as shame, sadness, stress and self-worthlessness. The consumption of cognitive resources would make them emotionally exhausted (Fernet et al., 2004), making it less likely for them to pay attention to focus on learning from failure during the innovation process.

Positive perfectionism is positively correlated to learning goal orientation in three ways. First, the nature of the innovation process as a recurring cycle of experimentation and learning means that (Thomke, 2003) only employees with positive attitudes toward the achievement of their goals, with emphasis on the achievement of favorable outcomes and motivated intrinsically, can learn adequately from failure (Danneels and Vestal, 2020; Hirst et al., 2009; Janssen and van Yperen, 2004), which are the key features of individuals high on positive perfectionism (Haase and Prapavessis, 2004). Second, setting higher goals usually means that they are more challenging to achieve, require higher effort, and are associated with lower chances of success than lower goals (Erez and Zidon, 1984). To achieve those higher goals, individuals need to increase learning and seek out creative activities during the innovation process (Hirst et al., 2009; Janssen and van Yperen, 2004). Third, employees with positive perfectionism tend to have more optimistic attitudes toward the achievement of their goals (Haase and Prapavessis, 2004) and place more emphasis on the feeling of fulfillment after achieving specific goals (Spector, 1956). They are more devoted to their learning and growing process and more focused on the enhancement of themselves (Dweck, 1986; Dweck and Leggett, 1988; Hirst et al., 2009; Janssen and van Yperen, 2004; Vande Walle, 1997), in contrast to solely focusing on the ultimate results of achieving their goals, which can increase their learning goal orientation. Thus, we propose the following hypotheses:

*Hypothesis 2a*: *Employees’ negative perfectionism is negatively related to their learning goal orientation.*

*Hypothesis 2b: Employees’ positive perfectionism is positively related to their learning goal orientation.*

### 2.4 Learning goal orientation and innovation performance

Innovation performance refers to the intentional generation, promotion, and realization of new ideas within a specific work role, work group, or organization to benefit role performance, a group, or an organization (Kanter, 1996; Scott and Bruce, 1994; West and Farr, 1989). During this process, learning goal orientation can be expected to be a vital motivational source for innovation performance for the following reasons (Hirst et al., 2009; Janssen and van Yperen, 2004).

First, learning goal orientation focuses employees’ attention on elaborating and developing new knowledge and “deep-processing” strategies that are effective in complex and unfamiliar tasks (Elliot and McGregor, 2001; Fisher and Ford, 1998; Steele-Johnson et al., 2000; Winters and Latham, 1996). Employees with a more potent learning orientation tend to be more intrinsically motivated to seek out creative activities that involve uncertain and untried approaches with a high possibility of error or potential failure (Ames and Archer, 1988; Hirst et al., 2009; Vande Walle, 1997). The strong tendency to pursue the tasks that are challenging and complex is an important feature of the innovation process, where employees are required to develop and apply their existing knowledge and requisite strategies to create something new (Janssen and van Yperen, 2004).

Second, employees with strong learning goal orientation are equipped with personal and intrinsic interests in the tasks that they are engaged in (Barron and Harackiewicz, 2000; Elliot, 1999; Pintrich, 2000c; Van Yperen, 2003). They are more likely to be motivated to put more efforts and innovate because of the pleasure accompanied by completing the tasks (Dweck, 1999; Janssen and van Yperen, 2004). They are willing to engage in the learning process of domain- and creativity-relevant skills (Hirst et al., 2009), which can provide the essential background knowledge and foundation for innovation and help them develop expertise (Amabile, 1996). When encountering obstacles, they have a strong intrinsic motivation to cope with the challenges by putting additional effort to develop and master new skills (Dweck, 1999; Vande Walle et al., 2001). The innovation literatures have found that the intrinsic motivation is essential for performing innovative activities (Amabile, 1988; Redmond et al., 1993), which can influence the extent to which employees are likely to put efforts to cope with the difficulties, respond to alternative ways, and thus become creative during engaging in the problem-solving tasks relevant to innovation (Amabile, 1988; Dweck, 1999; Hirst et al., 2009; Janssen and van Yperen, 2004). Thus, we propose the following hypothesis:

*Hypothesis 3: Employees’ learning goal orientation is positively associated with their innovation performance.*

By connecting hypotheses 1a and 1b, 2a and 2b, and 3, we propose the following hypothesis:

*Hypothesis 4: Employees’ learning goal orientation mediates the relationship between (a) employees’ negative perfectionism, (b) employees’ positive perfectionism, and their innovation performance.*

### 2.5 The moderating role of perceived failure analysis

Organizational cultures, defined as norms, values, and the practices that express them (Keith and Frese, 2011; van Dyck et al., 2005), vary in their responses to failure, and analysis is one of these cultures (Danneels and Vestal, 2020). Perceived failure analysis refers to the extent to which individuals within an organization believe that deliberate efforts are being made to examine the mechanisms and causes of past failures. This process typically involves conducting post-mortem reviews of previous decisions and carefully scrutinizing lessons learned from these experiences (Denrell, 2003; Edmondson, 2004; Tjosvold et al., 2004). Perceived failure analysis can provide an opportunity for firms to develop new knowledge that are crucial for their innovation capability (Calantone et al., 2002; Danneels, 2002; Maidique and Zirger, 1985), as better organizational knowledge can guide firms’ innovation choices and actions when faced with ambiguity (Danneels and Vestal, 2020). We argue that perceived failure analysis as an organizational culture facilitating firm innovation can influence the relationship between employee perfectionism and learning goal orientation because employees are embedded in the organizational environment and can be influenced by organizational culture (Goldberg et al., 2016).

Perceived failure analysis can strengthen the negative relationship between employee negative perfectionism and learning goal orientation for three reasons. First, a strong organizational culture that emphasizes failure analysis directly confronts employees with the detailed process of dissecting the mechanisms and causes of past failures (Denrell, 2003; Edmondson, 2004; Tjosvold et al., 2004). For employees with high negative perfectionism, this heightened focus on failure can amplify fear and avoidance tendencies due to the constant reminder of potential errors and the anticipated negative emotional impact of failure (Dunkley et al., 2003; Eggers, 2012; Haase and Prapavessis, 2004). This environment may discourage them from taking risks or experimenting and seeking out critical activities for learning and innovation, as it repeatedly reinforces their discomfort with failure (Shepherd et al., 2011; Stoeber and Otto, 2006). Second, a culture of failure analysis may lead employees to perceive that the high goals they set are often accompanied by a low likelihood of success (Erez and Zidon, 1984). This perception can diminish their motivation and commitment to achieving innovation-related goals (Locke and Latham, 2002). For perfectionistic employees who already doubt their ability to meet high standards, this perception of limited success may further discourage them from engaging in effortful learning activities, thus diminishing their learning goal orientation (Rubino et al., 2009; Skaalvik and Skaalvik, 2016). Third, frequent exposure to failure analysis can elicit repeated negative emotional responses, depleting cognitive resources that employees would otherwise dedicate to proactive learning (Fernet et al., 2004). For employees with high negative perfectionism, the added emotional and cognitive strain may contribute to emotional exhaustion, further diminishing their capacity and willingness to engage in learning activities aimed at overcoming and learning from failure.

Conversely, perceived failure analysis can strengthen the positive relationship between employee positive perfectionism and learning goal orientation for three reasons. First, a strong organizational culture that analyzes failure may provide employees with stronger intrinsic motivation to learn effectively from failure (Danneels and Vestal, 2020; Hirst et al., 2009; Janssen and van Yperen, 2004) because failure analysis can engage them into the process of dissecting the mechanisms and causes of past failures (Denrell, 2003; Edmondson, 2004; Tjosvold et al., 2004). This engagement can help them to better understand their goals in the future innovation process. Second, the setting of higher goals usually means that they are more difficult to achieve (Erez and Zidon, 1984), which means that they would experience the failure of achieving goals more frequently. A high organizational culture can help them better analyze their failures during this process, which can motivate them to put more efforts into increasing learning and seeking out creative activities during the innovation process (Hirst et al., 2009; Janssen and van Yperen, 2004). Third, employees with positive perfectionism are more likely to be devoted to their learning and growing process and are more focused on the enhancement of themselves (Dweck, 1986; Dweck and Leggett, 1988; Hirst et al., 2009; Janssen and van Yperen, 2004; Vande Walle, 1997), which means that they have better motivation to learn. While a high organizational culture that analyzes failure can provide better guidance for employees to learn, which can help them make progress more effectively during this process and satisfy their needs of self-enhancement, it can also strengthen the relationship between employee positive perfectionism and learning goal orientation. Thus, we propose:

*Hypothesis 5a: Perceived failure analysis moderates the negative relationship between employees’ negative perfectionism and their learning goal orientation, such that the negative relationship is stronger when failure analysis is high.*

Hypothesis 5b: *Perceived failure analysis moderates the positive relationship between employees’ positive perfectionism and their learning goal orientation, such that the positive relationship is stronger when failure analysis is high.*

### 2.6 Moderated mediation effects

According to the lens of achievement goal theory, a culture of failure analysis provides systematic feedback on errors, which enhances mastery-oriented individuals’ ability to convert failures into learning opportunities (Danneels and Vestal, 2020). For positively perfectionistic employees, this environment reinforces their mastery schema by validating effortful learning (Pintrich, 2000a), thereby strengthening the link between positive perfectionism and innovation via learning goal orientation. However, for negatively perfectionistic employees, failure analysis may exacerbate their performance-avoidance tendencies by highlighting the salience of errors (Elliot and Church, 1997), further depleting their motivation to engage in learning-oriented behaviors.

**Figure 1:**
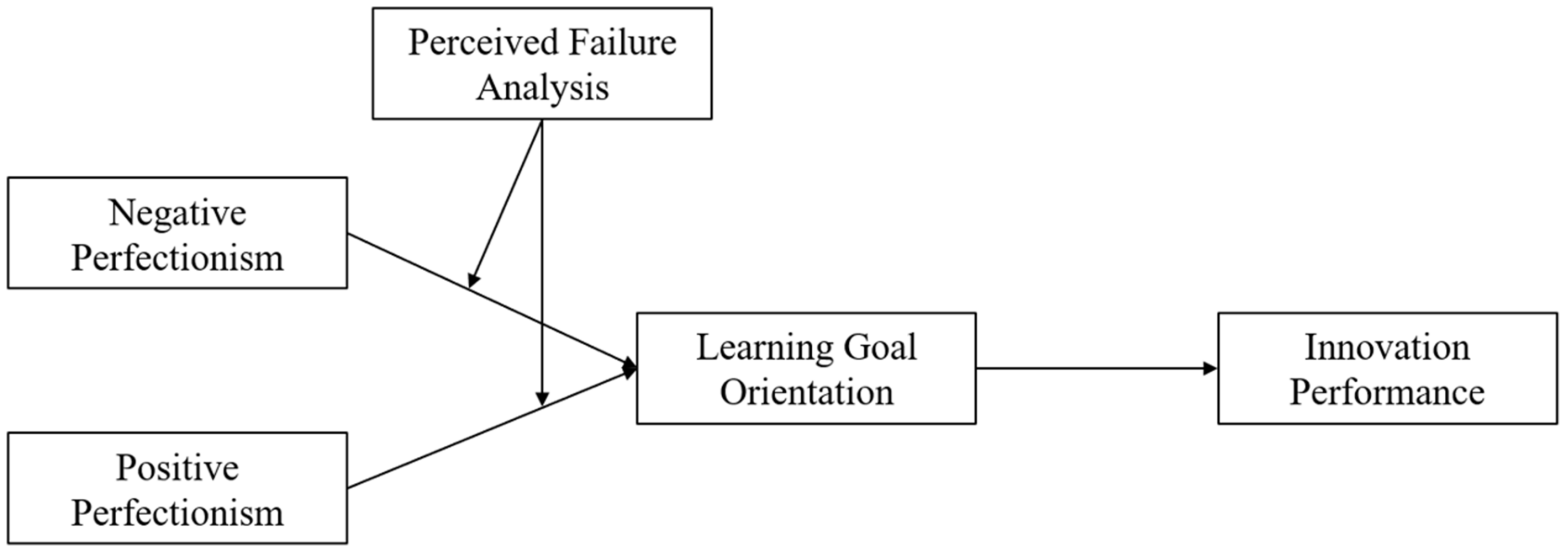
Research model.

Therefore, we propose the moderated mediation effects by connecting the mediation and moderating effects (Hayes, 2015; Preacher et al., 2007). When a moderator moderates the path of an otherwise simple mediation model, it is moderated by a moderator, and this effect is termed first-stage moderated mediation (Preacher et al., 2007). Following the similar logic, we have theorized (i) the mediated relationships of (a) negative perfectionism and (b) positive perfectionism on innovation performance through the mediating effect of learning goal orientation as well as (ii) the moderating effects of perceived failure analysis on the path from (a) negative perfectionism and (b) positive perfectionism to learning goal orientation. More specifically, we propose two first-stage moderated mediation effects in which perceived failure analysis moderates the indirect relationships between employee negative perfectionism and positive perfectionism on innovation performance through the mediating effect of learning goal orientation (Figure 1).

*Hypothesis 6a: Perceived failure analysis moderates the negative relationship between employees’ negative perfectionism and their innovation performance through the mediating effect of employees’ learning goal orientation, such that the negative mediated relationship is stronger when perceived failure analysis is high.*

*Hypothesis 6b: Perceived failure analysis moderates the positive relationship between employees’ positive perfectionism and their innovation performance through the mediating effect of employees’ learning goal orientation, such that the positive mediated relationship is stronger when perceived failure analysis is high.*

## 3 Methods

### 3.1 Sample

The participants are core research and development (R&D) employees from nine high-tech manufacturing companies across Shanghai, Jiangsu, Sichuan, Jilin provinces who specialized in the areas such as industrial internet-based automation equipment, intelligent architecture, information infrastructure building, and pharmaceutical products development. We first approached the top management teams from these companies and clarified our targeted respondents, the R&D employees serving their core business rather than the peripheral business. We were given personal information about 450 potential respondents, including their names, sex, age, education background, job, seniority, and their supervisors’ names. Referring to the employees’ job and seniority, we selected 413 suitable participants and allocated them with three-digit codes for data matching. Finally, we conducted a training session for all participants and selected a liaison agent from each company to help distribute questionnaires and encourage participants to finish questionnaires on time.

We collected data between 10 May 2021 and 15 June 2021, during which participants were surveyed in four consecutive waves, with an interval of 1 week for each round. In each data collection point, the correspondent agents of the firm received a link to the online questionnaires, and participants clicked the link distributed by the agents to fill in their answers. We collected data on our control variables in the first wave, including age, sex, educational background, managerial level, and consciousness. We collected data on the independent variables (i.e., negative perfectionism and positive perfectionism) and the moderator (i.e., perceived failure analysis) in the second wave; the data on the mediator (i.e., learning goal orientation) in the third wave; and data on the dependent variable (i.e., innovation performance) in the fourth wave.

We dropped unqualified samples according to the following standards: (1) based on responses to reverse-coded questions and the duration of the response time for further confirmation and (2) participants who did not answer the survey questions carefully, such as by using the same score to rate all items. After four waves of data collection and selection, 378 participants (91.5%) finished all waves of questionnaires and provided eligible answers. Hence, our final sample (with no missing data for our key research variables) consists of 378 respondents. The final sample for hypotheses testing is composed of 82.08% male and 17.92% female individuals. Their ages range from 24 to 56 years, with an average age of 34.03 years. Married participants accounted for 74.26%. Approximately 62.50% of the participants have a bachelor’s degree, and 20.59% of the participants have a master’s degree. The percentage of employees and managers is 72.30 and 27.69%, respectively. In terms of participants’ length of employment, the longest is more than 30 years while the shortest is less than 1 year, with an average of 7.41 years. In general, the sample is representative of the population in which our research is focused on.

### 3.2 Measures

The original scales were in English and translated into Chinese when distributed to our participants. We followed a back-translation procedure (Brislin, 1980) to ensure consistency between the Chinese and English items. A 5-point Likert scale was used to measure our theoretical model (excluding control variable), and survey participants provided their responses on a scale ranging from 1 (strongly disagree) to 5 (strongly agree).

#### 3.2.1 Negative and positive perfectionism

We used the 19-item scale revised by Haase and Prapavessis (2004) to measure respondents’ negative perfectionism and positive perfectionism. Sample items of negative perfectionism are “If I fail people, I fear they will cease to respect or care for me” and “I feel I have to be perfect to gain people’s approval.” Additionally, sample items of positive perfectionism are “I enjoy working towards greater levels of precision and accuracy” and “I feel good when pushing out the limits.” The Cronbach’s alpha of positive perfectionism and negative perfectionism are 0.89 and 0.90, respectively.

#### 3.2.2 Learning goal orientation

We employed the 4-items scale designed by Vande Walle et al. (2001) to measure respondents’ learning goal orientation. Sample items are “I prefer challenging and difficult classes so that I’ll learn a great deal” and “I truly enjoy learning for the sake of learning.” The Cronbach’s alpha for the scale is 0.87.

#### 3.2.3 Perceived failure analysis

We measured perceived failure analysis using the 5-items inventory by Danneels and Vestal (2020). Sample items are “We review past decisions, especially if they did not lead to success” and “We go to great lengths to learn from failures,” The Cronbach’s alpha for the scale is 0.95.

#### 3.2.4 Innovation performance

We measured innovation performance using the 9-item scale designed by Janssen (2001). Sample items are “I can create new ideas for improvements” and “I introduce innovative ideas in a systematic way.” We asked respondents to self-report their innovative activities in the workplace, and the Cronbach’s alpha is 0.98. Janssen (2001) reported that respondents’ self-reports on their innovative performance are more “subtle” than the reports from their supervisors, and the common rater bias is not a primary concern. Supervisors usually notice the activities intended to draw their attention (Organ and Konovsky, 1989). Thus, employees’ innovation performance, rated by supervisors, reflects employees’ ability to signal their achievement to others rather than employees’ ability to make differences in their workplaces.

#### 3.2.5 Control variables

We controlled for respondents’ sex (female = 0; male = 1), age (in years), education background (high school or below = 1, bachelor’s degree = 2, and master’s degree = 3, doctor = 4), and managerial level (entry level = 1, senior manager = 4) because they may influence people’s innovative performance (Gong et al., 2009). We also controlled for respondents’ consciousness, which may prompt people to devote more efforts into their work, influencing their performance in workplaces. A participant’s consciousness is measured by the short version of Big Five Inventory designed by Rammstedt and John (2007). Participants first read two statements “I usually do a thorough job” and “I tend to be lazy” and then choose from 1 to 5 to demonstrate the extent to which the two statements reflect their personality.

## 4 Analysis and results

### 4.1 Confirmatory factor analysis

The descriptive statistics and correlations are displayed in Table 1. Using Mplus 8^5^ (Muthén and Muthén, 2010), we performed a series of confirmatory factor analyses on the measurement items of negative perfectionism, positive perfectionism, learning goal orientation, perceived failure analysis, and innovation performance. The five-factor model provided an adequate fit: *χ*^2^ = 1936.01, *df* = 619; comparative fit index (CFI) = 0.90; standardized root mean square residual (SRMR) = 0.08. This model was compared to an alternative model that combined negative perfectionism and positive perfectionism into one factor, as they were reported by the same individuals at the same time. This three-factor model provided a poor fit: *χ*^2^ = 3,556.56, *df* = 623; CFI = 0.77; SRMR = 0.16. A chi-squared difference test showed that it fitted significantly worse than the four-factor model: Δ*χ*^2^ = 1620.55, Δ*df* = 4, *p* < 0.01. This model was also compared with an alternative model in which learning goal orientation and perceived failure analysis were combined into one factor for the similar reason. This three-factor model provided a poor fit: χ^2^ = 2,729.95, *df* = 623; CFI = 0.83; SRMR = 0.13. A chi-squared difference test showed that it fitted significantly worse than the four-factor model: Δ*χ*^2^ = 793.94, Δ*df* = 4, *p* < 0.01.

**Table 1:** Descriptive statistics and pairwise correlations.

Variables	Mean	SD	1	2	3	4	5	6	7	8	9	10
1. Innovation performance	3.56	0.85	(0.98)									
2. Negative perfectionism	2.72	0.80	−0.16	(0.90)								
3. Positive perfectionism	4.33	0.58	0.44	−0.11	(0.89)							
4. Learning goal orientation	4.04	0.67	0.59	−0.22	0.55	(0.87)						
5. Perceived failure analysis	4.43	0.69	0.36	−0.17	0.35	0.43	(0.95)					
6. Age	33.73	6.09	0.01	0.03	−0.00	−0.01	−0.08	−				
7. Sex	0.19	0.40	−0.07	−0.10	−0.11	−0.09	0.06	0.04	−			
8. Education background	2.08	0.66	0.05	−0.12	−0.08	−0.00	−0.02	0.05	0.14	−		
9. Managerial level	2.11	0.84	0.08	0.01	0.14	0.04	0.00	0.39	0.07	0.16	−	
10. Consciousness	4.08	0.75	0.30	−0.18	0.30	0.38	0.44	−0.01	0.03	−0.01	0.05	−

*N = 378.*

Cronbach’s alpha coefficients are shown in brackets.

Correlations greater than 0.10 or less than − 0.10 are significant when the *p*-value is < 0.05.

### 4.2 Test of hypotheses

**Figure 2:**
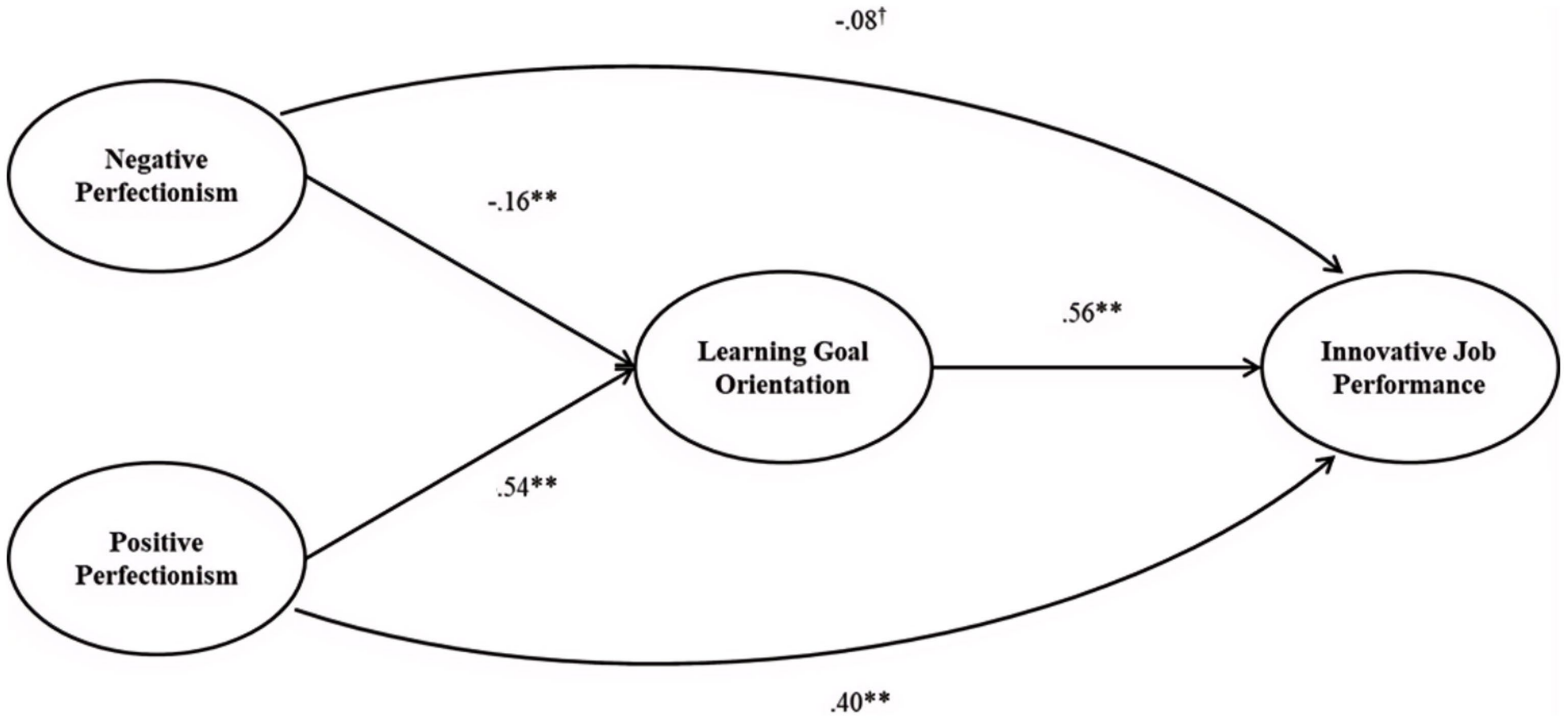
SEM results. ^†^*p* < 0.1, *p < 0.05, **p < 0.01, and ***p < 0.001.

In addition to testing the measurement adequacy in obtaining empirical data, we also used Mplus 8 to analyze our hypotheses with the empirical data. We followed the recommendations by Holland (1988) to employ structural equation modeling (SEM) to test the mediation effect of employees’ learning goal orientation on the relationship between the two types of their perfectionism and innovation performance. Figure 2 shows the model results. Hypothesis 1a posited a direct negative relationship between negative perfectionism and innovation performance, while hypothesis 1b posited a direct positive relationship between innovation performance and positive perfectionism. The result supports hypothesis 1a, whose direct negative effect is marginally significant (*β* = −0.08, *p* < 0.10). The result also supports hypothesis 1b, whose direct positive effect is significant (*β* = 0.40, *p* < 0.01).^1^

Hypothesis 2a posited that employees’ negative perfectionism is negatively associated with the extent of their learning goal orientation, and hypothesis 2b predicted that employees’ positive perfectionism is positively associated with the extent of their learning goal orientation. Both hypotheses are confirmed by our SEM results shown in Figure 2. There is a significantly negative relationship between negative perfectionism and learning goal orientation (*β* = −0.16, *p* < 0.01) and a significantly positive relationship between positive perfectionism and learning goal orientation (*β* = 0.54, *p* < 0.01).

Hypothesis 3 stated that employees’ learning goal orientation is positively correlated with innovation performance. Furthermore, this hypothesis is supported by the model results shown in Figure 2, where a significantly positive relationship exists between learning goal orientation and innovation performance (*β* = 0.56, *p* < 0.01).

By integrating hypotheses 1a and 1b as well as 2a and 2b into s 3, hypothesis 4 stated the mediation effect that employees’ learning goal orientation mediates the relationship between employees’ negative and positive perfectionisms and their innovation performance. To test the indirect effects in our model more clearly, we determined the significance of our mediation effect by using bootstrapped 95% confidence intervals (CI) with 20,000 repetitions (Bollen and Stine, 1990). The indirect effect of employees’ negative perfectionism on their innovation performance via their learning goal orientation was significantly negative (*β* = −0.08, 95% CI = [−0.13, −0.03]). The indirect effect of employees’ positive perfectionism on their innovation performance via learning goal orientation was significantly positive (*β* = 0.37, 95% CI = [0.27, 0.48]). Therefore, hypothesis 4 is supported.

In the set of hypotheses 5a and 5b, we argued that perceived failure analysis strengthens not only the negative relationship between employees’ negative perfectionism and their learning goal orientation but also the positive relationship between employees’ positive perfectionism and their learning goal orientation. The results indicate that an interaction between employees’ negative perfectionism and perceived failure analysis have no significant impacts on employees’ learning goal orientation (*β* = −0.10, *p* > 0.10). In addition, the interaction between employees’ positive perfectionism and perceived failure analysis culture significantly and positively indicates employees’ learning goal orientation (*β* = 0.18, *p* < 0.05). Therefore, hypothesis 5b is supported, while hypothesis 5a is not supported.

Our final set of hypotheses predict a moderation effect of a typical organizational culture on the prescribed main effect. Hypothesis 6a posited that the organization culture, as it pertains to analyzing failure, would strengthen the negative indirect effect of employees’ negative perfectionism on their innovation performance. Hypothesis 6b stated that analyzing failure within the organizational culture would strengthen the positive indirect effect of employees’ positive perfectionism on their innovation performance. We adopted two methods to probe these two first-stage moderated indirect effects.

**Figure 3:**
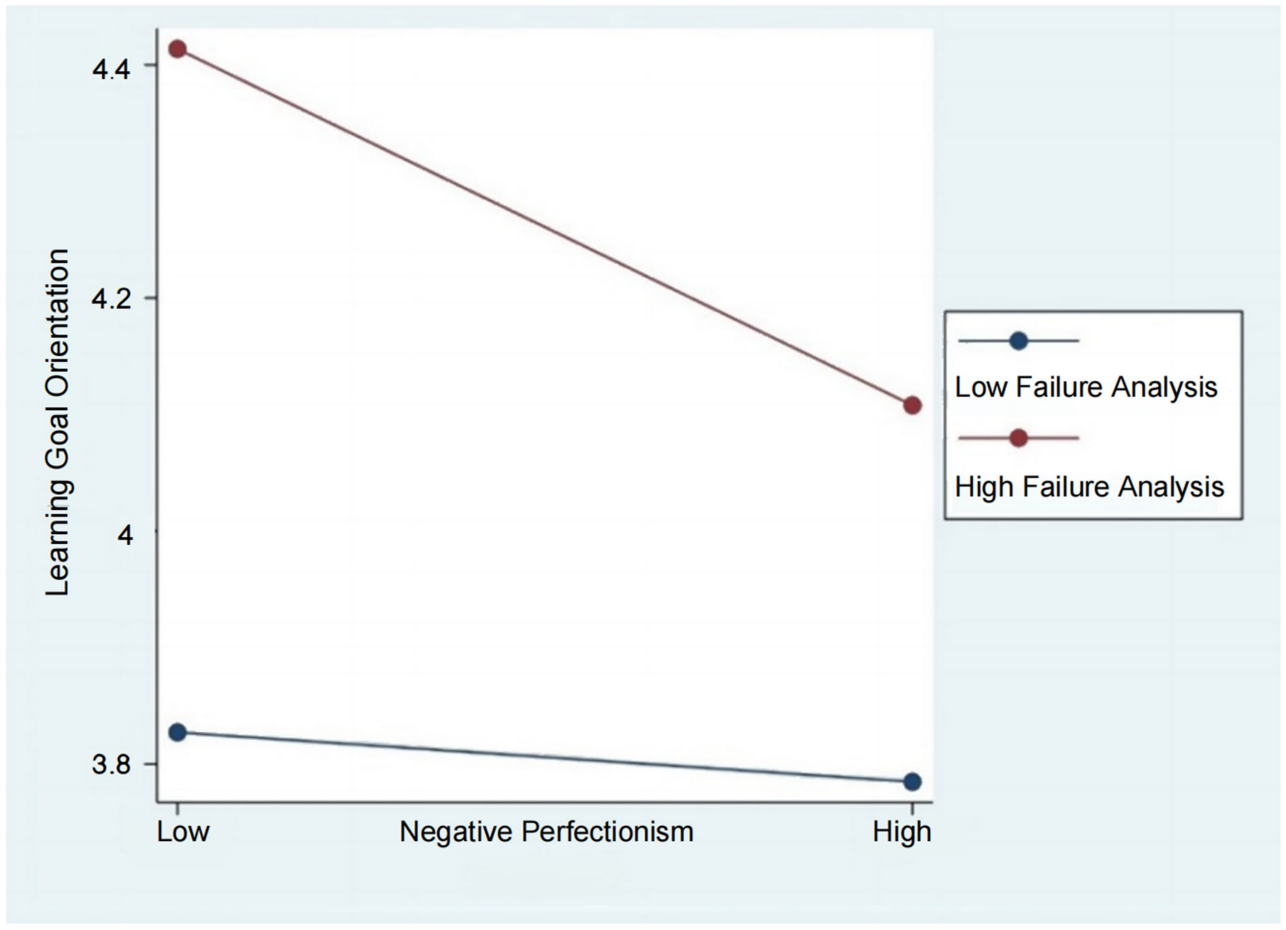
The moderating effect of perceived failure analysis on the relationship between positive perfectionism and learning goal orientation.

**Figure 4:**
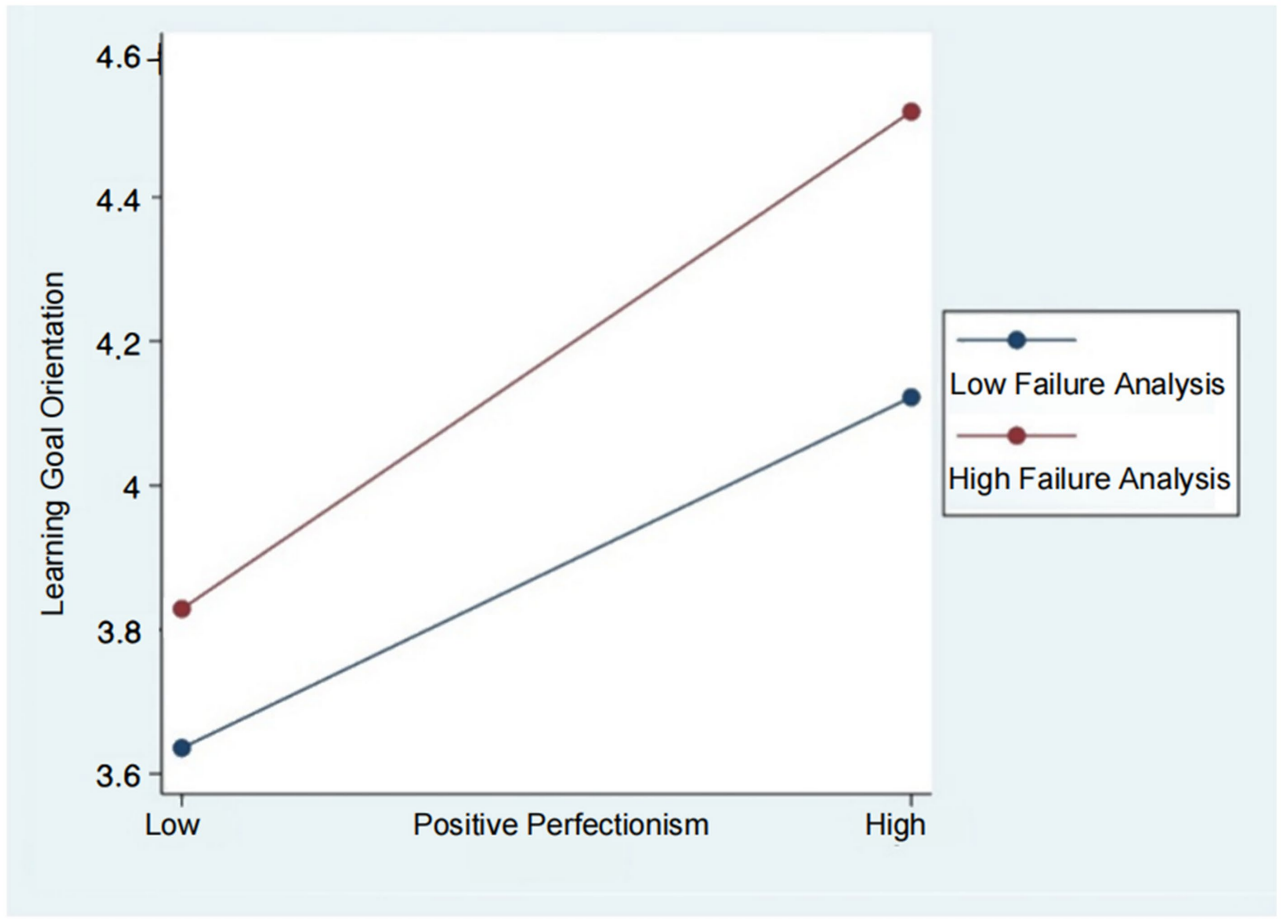
The moderating effect of perceived failure analysis on the relationship between positive perfectionism and learning goal orientation.

First, we followed the suggestion from Dawson (2014) to examine the interactions visually by using the simple slopes test. We plotted the interaction across organizations with strong perceived failure analysis (one standard deviation above the mean) and weak perceived failure analysis (one standard deviation below the mean). As shown in Figure 3, the effect of employees’ negative perfectionism on their innovation performance is more negative when perceived failure analysis is stronger when perceived failure analysis is high compared to when it is low. Similarly, in Figure 4, we plotted the interactions of employees’ positive perfectionism and their innovative performance across the two different organizational cultures. Figure 4 shows that, for employees’ innovative performance, positive perfectionism was a more significant positive predictor in organizations with strong perceived failure analysis compared to those with weak perceived failure analysis. However, it remains unclear if the difference is significant, and further comparisons between these two groups are necessary.

Second, we followed the approach of Edwards and Lambert (2007) and performed a simple indirect effects analysis across the three levels of perceived failure analysis (mean + standard deviation [M + 1 SD], M, and M – 1 SD). We calculated the moderated indirect effect in path 1 as the product of the estimated joint effect of employees’ negative perfectionism and perceived failure analysis, along with the estimated effect of employees’ learning goal orientation on their innovation performance. The results indicate that the difference between the simple indirect effects at high- and low-level perceived failure analyses is not significant (*β* = 0.08, 95% CI = [−0.19, 0.00]), suggesting that hypothesis 6a is not supported (Table 2).

**Table 2:** Analysis of moderated indirect effects.

	Innovation performance		
	Effect	90% CI	95% CI
Moderated indirect effect (Path 1: negative perfectionism→learning goal orientation→innovation performance)			
Simple indirect effect at high perceived failure analysis (M + 1 SD)	−0.09	[−0.14, −0.05]	[−0.16, −0.04]
Simple indirect effect at mediate perceived failure analysis (M)	−0.06	[−0.10, −0.02]	[−0.11, −0.02]
Simple indirect effect at low perceived failure analysis (M – 1 SD)	−0.02	[−0.08, 0.04]	[−0.09, 0.06]
Difference between the simple indirect effects of high-level and low-level perceived failure analysis	-.08^a^	[−0.16, −0.01]	[−0.19, 0.00]
Moderated indirect effect (Path 2: positive perfectionism→learning goal orientation→innovation performance)			
Simple indirect effect at high perceived failure analysis (M + 1 SD)	0.39	[0.29, 0.51]	[0.27, 0.54]
Simple indirect effect at mediate perceived failure analysis (M)	0.31	[0.24, 0.40]	[0.23, 0.42]
Simple indirect effect at low perceived failure analysis (M – 1 SD)	0.24	[0.16, 0.33]	[0.15, 0.34]
Difference between the simple indirect effects of high-level and low-level perceived failure analysis	.14^b^	[0.05, 0.28]	[0.03, 0.30]

^aThe value difference is caused by rounding up.^

Similarly, we calculated the moderated indirect effect in path 2 as the product of the estimated joint effect of employees’ positive perfectionism and their perceived failure analysis, along with the estimated effect of employees’ learning goal orientation on their innovation performance. The results indicate that the difference between the simple indirect effects at high- and low-level perceived failure analyses is significant (*β* = 0.14, 95% CI = [0.03, 0.30]), suggesting that hypothesis 6b is supported (Table 2).

## 5 Discussion

### 5.1 Theoretical implications

This study advances the literature on perfectionism and innovation by integrating achievement goal theory with a dual-dimensional framework of perfectionism, presenting three key theoretical contributions.

First, we resolve a critical ambiguity in previous research by demonstrating *how* the multidimensional nature of perfectionism differentially influences innovation through distinct motivational pathways. While earlier studies often treated perfectionism as a unidimensional trait linked to stress or burnout (Childs and Stoeber, 2012; Harari et al., 2018), our findings align with achievement goal theory (Pintrich, 2000a; Elliot and McGregor, 2001) to propose that positive perfectionism operates as a mastery-oriented schema that fosters a learning goal orientation (e.g., embracing challenges and persisting through setbacks), whereas negative perfectionism reflects a performance-avoidance schema that prioritizes error prevention over exploration. This distinction clarifies why positive perfectionism enhances innovation (via proactive learning), while negative perfectionism stifles it (via risk aversion)—a paradox that has been overlooked in previous studies (Ozbilir et al., 2015). By grounding perfectionism’s duality in achievement goal theory, we bridge the perfectionism-innovation literature with motivational psychology, providing a unified framework to explain conflicting empirical findings.

Second, we enhance achievement goal theory by identifying perceived failure analysis as a vital cultural moderator that amplifies the benefits of mastery-oriented perfectionists. While Danneels and Vestal (2020) emphasized failure analysis as a driver of organizational learning, our study reveals its nuanced role in individual motivation: failure analysis strengthens the link between positive perfectionism and innovation by validating mastery goals (e.g., framing failures as learning opportunities). However, it fails to mitigate the harmful effects of negative perfectionism because avoidance-oriented employees perceive such analysis as threatening rather than informative (Elliot and Church, 1997). This finding advances the theory by demonstrating that organizational practices should align with employees’ motivational orientations to unlock innovation potential—a boundary condition that has been previously underexplored.

Third, our integration of perfectionism into achievement goal theory responds to calls for contextualizing personality traits within organizational settings (Harari et al., 2018). By conceptualizing perfectionism as a *motivational lens* that influences how employees interpret and tackle challenges, we enhance the explanatory power of achievement goal theory. For instance, our results suggest that personality traits like perfectionism may predispose individuals to adopt specific goal orientations (e.g., mastery vs. avoidance), mediating their innovation behaviors. This finding builds on previous research study focused on situational antecedents of goal orientations (Pintrich, 2000b) and establishes a foundation for future research to explore trait–context interactions.

### 5.2 Limitations and future research

As with any research, we do not believe that our findings are immune to potential limitations. First, there are different types of goal orientation in addition to learning goal orientation (Vande Walle et al., 2001). For simplicity and clarity, this research primarily focuses on the intrinsic motivation of perfectionistic employees to innovate, while extrinsic motivation may also impact their innovation performance. The results might be more interesting when we consider both intrinsic and extrinsic motivation.

Second, although we employed a multiwave data collection method to mitigate common method bias, it remains a potential concern. On the contrary, all variables were measured through self-reports, which may lead to inflated correlations due to shared method variance. Future research can also use objective criteria to assess employees’ innovation performance as a robustness test of the relationships in our study. Conversely, despite the time-lagged design involving a 1-week interval between each wave, respondents’ responses may still be influenced by common factors such as response styles, social desirability bias, or mood states. Future research should explore more diverse data collection methods, such as Experience Sampling Method (ESM), to reduce the impact of common method bias.

Third, our study assumes positive and negative perfectionism as separate constructs; however, Hill et al. (2004) point out that individuals may exhibit both adaptive and maladaptive tendencies. While our model considers their statistical independence, future research should explore interactive effects (e.g., high positive and high negative perfectionism) using multidimensional measures like the Perfectionism Inventory. Such studies could reveal whether “mixed perfectionists” exhibit unique innovation patterns, such as high effort combined with anxiety-driven risk aversion.

## 6 Conclusion

This study presents a novel and clear theoretical explanation of employee innovation performance by unraveling the intervening mechanism of the double-edged sword effect of employee perfectionism on their learning goal orientation. This explanation (a) highlights and distinguishes the multidimensional characteristics of perfectionism and its different impacts on innovation performance through the mediating effect of learning goal orientation; (b) emphasizes the importance of learning during the innovation process by introducing the concept of learning goal orientation as a mediator; and (c) considers perceived failure analysis as a part of organizational culture and discusses its impacts on the aforementioned relationships between employee perfectionism, learning goal orientation, and innovation performance. We hope our discussion of employees’ innovation performance, based on their motivational processes, will offer insights to further research on perfectionism, innovation, and achievement goal theory.

## Data Availability

The raw data supporting the conclusions of this article will be made available by the authors, without undue reservation.

## Footnotes

^5^ https://www.statmodel.com/index.shtml

^1^We controlled for respondents’ sex, age, education background, and managerial level when we tested the hypothesis. The effects of all control variables on people’s innovation performance are not significant (*p* > 0.05).
